# Zika Virus Potentiates the Development of Neurological Defects and Microcephaly: Challenges and Control Strategies

**DOI:** 10.3389/fneur.2019.00319

**Published:** 2019-04-09

**Authors:** Rabeea Siddique, Yang Liu, Ghulam Nabi, Wasim Sajjad, Mengzhou Xue, Suliman Khan

**Affiliations:** ^1^Henan Medical Key Laboratory of Translational Cerebrovascular Diseases, Zhengzhou University, Zhengzhou, China; ^2^The Department of Cerebrovascular Diseases, The Second Affiliated Hospital of Zhengzhou University, Zhengzhou, China; ^3^The Key Laboratory of Aquatic Biodiversity and Conservation of Institute of Hydrobiology, Chinese Academy of Sciences, Wuhan, China; ^4^University of Chinese Academy of Sciences, Beijing, China; ^5^Key Laboratory of Petroleum Resources, Gansu Province/Key Laboratory of Petroleum Resources Research, Institute of Geology and Geophysics, Chinese Academy of Sciences, Lanzhou, China

**Keywords:** Zika virus, emerging infection, microcephaly, neurological disorders, public health

## Abstract

Since the beginning of the Zika Virus (ZIKV) epidemic, thousands of cases presenting ZIKV symptoms were recorded in Brazil, Colombia (South America), French Polynesia and other countries of Central and North America. In Brazil, during ZIKV outbreak thousands of microcephaly cases occurred that caused a state of urgency among scientists and researchers to confirm the suspected association between ZIKV infection and microcephaly. In this review article we comprehensively studied scientific literature to analyze ZIKV relationship with microcephaly, recent experimental studies, challenge and shortcomings in previously published reports to know about the current status of this association. The evidences supporting the association of ZIKV infection with congenital microcephaly and fetal brain tissue damage is rapidly increasing, and supplying recent information about pathology, clinical medicine, epidemiology, mechanism and experimental studies. However, serious attention is required toward ZIKV vaccine development, standardization of anthropometric techniques, centralization of data, and advance research to clearly understand the mechanism of ZIKV infection causing microcephaly.

## Introduction

**Figure 1:**
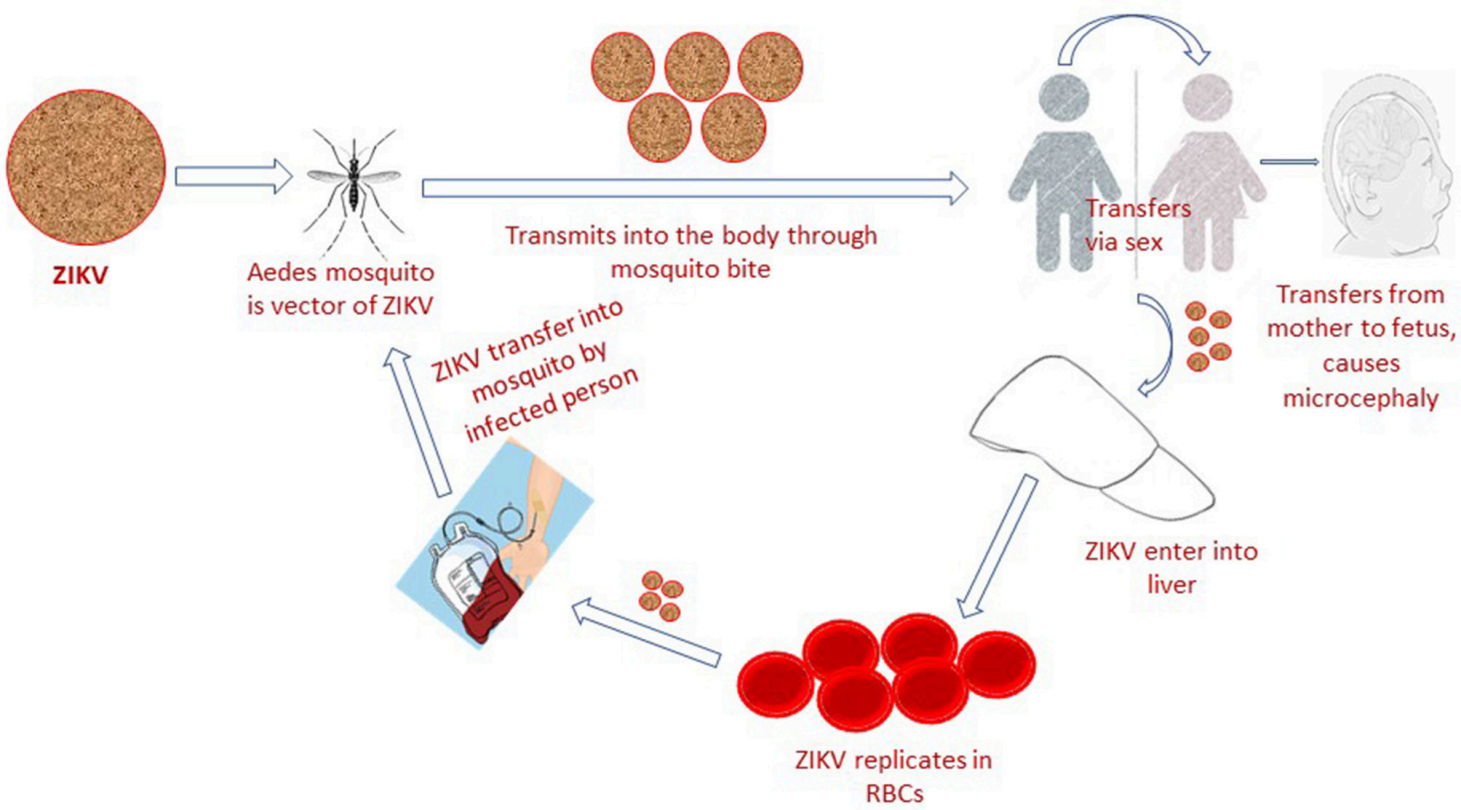
Zika virus infection is transmitted to human population via mosquito bite, blood transfusion, sexual intercourse, and from mother to fetus.

The Zika virus (ZIKV) is a mosquito-borne, single-stranded RNA, flavivirus that is closely related to yellow fever virus (YFV), Japanese encephalitis virus (JEV) and dengue virus (DENV). It can adapt to harsh conditions and temperature as high as 40°C (1, 2). ZIKV natural transmission cycle mainly involves the genus *Aedes* mosquito species (*A. furcifer, A. luteocephalus, A. africanus, and A. taylori*), sylvatic cycle (monkeys) and occasional human hosts (3). In human population it is potentially transmitted via sex (heterosexual or homosexual transmission) (4), blood transfusion (5), from mother to fetus and through direct contact (6) (Figure 1). The most common laboratory tests for Zika virus confirmation constitute both RNA and antibody detection in plasma, urine, amniotic fluid, conception products, autopsy and placental tissue (7), cerebrospinal fluid, semen, saliva and breast milk, which confirm the above-mentioned routes of transmission (8). Based on lack of standardized diagnostic test facilities, non-elimination of other confounding factors and ambiguities in previously reported data (9), the ZIKV association with microcephaly was considered ambiguous. However, the World Health Organization situation report based on observational, cohort and case-control studies claimed a strong scientific consensus that ZIKV infection is cause of microcephaly, Guillain-Barré syndrome (GBS) and other congenital neurological disorders (10). A recent Lancet report has also presented genuine facts that ZIKV infection is the cause of congenital microcephaly (11).

The geographically widespread epidemic of ZIKV emerged as a profoundly important public health concern when congenital microcephaly and other fetal/neonatal abnormalities were recorded in infected pregnant women (12). Congenital microcephaly is a rarely occurring neurological birth defect, characterized by fetal head circumference (HC) at least 2 standard deviations (SD) below the mean size of other fetuses/babies at the same gestational age (13), sex and ethnicity; and if HC is at least 3 SD smaller than it is considered severe (14). Microcephaly may occur alone or with other congenital malformations, such as prognosis of intellectual and/or motor disabilities, including speech retardation, physical disability (13), behavioral issues, and poor neurocognitive outcomes (9). Due to its complex mechanisms and multifaceted etiology still there is no universally accepted and uniform diagnostic standard of microcephaly (15). The possible etiologies may include environmental or genetic factors during pregnancy, such as, perinatal brain injury, craniosynostosis, drugs, hypertensive disorders, intrauterine infections caused by West Nile virus (WNV), and Chikungunya virus (CHIKV) (9), and other prenatal viral infections e.g., cytomegalovirus, syphilis, rubella, herpesvirus, and toxoplasmosis (TORCHES) (7). Intriguingly, it was not included in the list of neurological infection causing microorganisms until 2015 (7, 16).

The number of suspected congenital microcephaly cases associated with ZIKV in Brazil had increased to 2975 by January 2016, and new cases of adult ZIKV infection were being reported from countries throughout the Caribbean and Central and South America (12). The World Health Organization (WHO) declared ZIKV as Public Health Emergency of International Concern (PHEIC) on February 2016, when recorded congenital microcephaly cases soared up in the geographically widespread ZIKV epidemic regions (7, 16, 17), causing a state of urgency among scientists to find out its association with ZIKV, initially known to cause febrile illness (17).

ZIKV-induced malformations require serious attention and consideration because core information about its pathogenicity and mechanism of action is either obscure or evolving (17). The purpose of this review article is to comprehend if ZIKV can potentially cause microcephaly. For this purpose, we comprehensively accessed and characterized current literature to discuss about the widespread epidemic of ZIKV, pathology of ZIKV related microcephaly, recent *in vitro* and *in vivo* experimental studies to understand the mechanism of ZIKV infection, ambiguities in previously published data and ZIKV future implications, its control strategies and hints of advance research to affirm this probationary link.

## ZIKA Virus Epidemics

The ZIKV was first discovered and isolated from the blood of a sentinel rhesus monkey in the Zika forest of Uganda, in 1947 (18). It was occasionally found in Africa and Asia since its epidemic occurred in the Yap Island, the Federal state of Micronesia (2007), French Polynesia (2013), Colombia (12), and Brazil (2015) (7, 19). As per estimation, from 2007 to 2016, ZIKV outbreak has affected several regions of America, Africa, Southeast Asia (20), Caribbean and western pacific island (21). In the United States, ZIKV cases have been reported in American Samoa, US Virgin Islands and Puerto Rico. In USA, the first ZIKV associated congenital microcephaly case was reported in Hawaii, in 2016 (21). Due to a high cross-reactivity rate of ZIKV it is often misdiagnosed as DENV as happened in the Yap Island. Its potential association with microcephaly has been reported in the French Polynesia, Brazil, America, Slovenia and Colombia (22).

During the Zika virus pandemic in the French Polynesia, an unexpected rise in the number of autoimmune and neurological complications was observed. Almost 66% (95% CI 62–70) of the general population developed infection and more than 31,000 patients consulted physicians due to suspected infection (9). Among patients presenting ZIKV like symptoms that visited health care units, 1.3 per 1,000 (42 cases) had GBS, while, 2.3 per 1,000 had neurological complications (16). From 2014-15, Brazil reported multiple cases of illness and skin rash to the World Health Organization (WHO), occurring in the Brazilian states Pernambuco, Maranha~o, Rio Grande do Norte and Bahia. In these states (February to April 2015), around 7,000 skin rash cases were reported but no identification tests were performed as ZIKV infection was not suspected (12). After the ZIKV epidemic, 18 of the 27 states of Brazil reported ZIKV autochthonous cases between April and November 2015. It caused a 20-fold increase in the microcephaly cases in Brazil, whereas, around 1,248 new suspected cases were observed, a prevalence of 99·7/100,000 live births. The WHO issued an epidemiological alert about the relationship of ZIKV infection with neurological syndromes and congenital microcephaly, after confirmation by Brazilian Ministry of Health (11). In the Brazilian state, Bahia, multiple cases of acute rash were followed by increased number of microcephalic fetuses (13), while in Pernambuco, almost 2% of all the symptomatic and asymptomatic mothers were suspected to be diagnosed with microcephalic fetuses. Only half of the reported cases were further confirmed by the presence of calcification, other brain malformations, or both (23). According to routine birth reports, an average of 163 (5.6 per 100,000 live birth) cases of microcephaly occurred annually before 2015 ZIKA epidemic in Brazil, while 3,530 (121.7 per 100,000 live births) suspected cases of microcephaly were reported in 2015, including 46 death cases (16). It is estimated that in South America the ZIKV epidemic has infected more than 1 million Brazilians until 2015 (24).

## Clinical Manifestations of ZIKV Infection

Pathology studies have significantly contributed to the understanding of fetal/neonatal anatomic abnormalities and relationship between intrauterine ZIKV infection and damage to cerebral tissue (12). Here we discussed ZIKV related microcephaly clinical manifestations to better understand the outcomes of this infection on pregnant women and fetus/neonates.

A retrospective review (March 2014–May 2015), was conducted to identify ZIKV related brain abnormalities in French Polynesian neonates. It was reported that 8 out of 19 identified cases had severe microcephaly with major brain lesions, five had visible malformations and cerebral dysfunction, while six had brain lesions without microcephaly. The imaging results showed acute neurological lesions (including septal and colossal disruption, abnormal neuronal migration, cerebellar hypoplasia), and brain calcifications (9). Another coinciding report on laboratory-confirmed microcephalic infants and fetus autopsies had shown viral-induced, visible microscopic brain deformations including, degenerative changes in neuronal and glial cells, gliosis, necrosis, white matter, and axonal rarefaction decline, microcalcifications and viral-induced cytopathic effects (25, 26). The ZIKV-RNA detected from amniotic fluid of two Brazilian infected mothers whose blood samples were negative for virus (13), showed that ZIKV can possibly cross the placental barrier (27).

Cauchemez et al. calculated the risk of microcephaly during ZIKV outbreak in French Polynesian outbreak (Sept, 2013, to July, 2015) through statistical data, mathematical models and serologic data (9). The authors has suggested that 95 out of 10,000 cases (1%) of neonates and fetuses whose mothers got ZIKV infection in their first trimesters had microcephaly, a prevalence that was around 50 times as high as the calculated baseline prevalence. Based on the findings of computed tomography (CT), 23 cases of microcephalic infants born to mothers showing ZIKV symptoms during first or second trimesters were discussed by authors (28). While another report presented 22% risk of microcephaly during first trimester. Similarly, a more recent cohort study has reported that prevalence of ocular and neurologic malformations were more commonly observed during the first trimester of ZIKV infected mothers (12.7%) as compared to second (3.6%) and third trimester (5.3%) (*P* = 0.001) (29).

The fetus brain postmortem report by Mlakar et al. (30) indicated the presence of flavivirus-like particles (viral RNA load, 6.5 × 10^7^ copies per mg), hydrocephalus, several microscopic malformations, focal inflammation, calcifications, displacement of cortex, and HC below then second percentile. While phylogenetic analysis has revealed the highest resemblance ratio (99.7%) with French Polynesia, Sao Paolo, Brazil, Cambodia, and Micronesia ZIKV strain (30). Similarly, cerebral examination of two microcephalic neonates and two e autopsies exposed multiple pathologic findings, such as, microglial nodules, gliosis, cellular degeneration, parenchymal calcifications, and necrosis. Here all reported mothers were symptomatic for ZIKV infection during the first trimesters of their pregnancy and RT-PCR gave positive results for ZIKV RNA in the brain and placental tissue samples (12). The ZIKV intrauterine infection cases during third trimester, were also found associated with reduction in brain parenchymal volume and corpus callosum development (13). In another fetal case report, HC declined from 47th to 24th percentile without microcephaly and intracranial calcifications (15). The infant's cerebrospinal fluid sample analysis by enzyme-linked immunosorbent assay (ELISA) gave positive Ig-M antibody result, while occurrence of white -matter dysmyelination and cortical hypogyration was claimed to be caused by ZIKV infection as it attenuates the brain development (28).

The general purpose of amniotic fluid and fetal brain tests is to know if the virus can get through the placental barrier; microscopic placental examination to identify focal chorionic and/or calcific villi (30), and ultrasonography to get abnormal placental image (31). Amniocentesis for ZIKV detection, on the contrary, is largely suggested especially in case of asymptomatic fetuses despite lack of information about ZIKV predictive positive value and amniotic fluid molecular detection (31).

## The Mechanism of ZIKV-Mediated Brain Damage

Laboratory testing, *in vivo* animal models and *in vitro* cellular systems are profoundly important to understand and explain the cellular and molecular basis of microcephaly development, level of selective neurotropism (25), spectrum of changes occurring to the brain, and pathophysiology of viremia in ZIKV infected fetuses (27). Recently conducted animal experiments have confirmed that ZIKV can cause fetal demise, hydropsfetalis (32), and placental damage (33), by destroying central nervous system and causing severe pathological changes (27).

### ZIKV Related *in-vivo* Studies

Li et al. (34) used a contemporary ZIKV strain on a mouse model to predict the ZIKV relationship with microcephaly. Their findings suggested that ZIKV effectively replicated in embryonic mouse brain and caused viral induced apoptosis, cell-cycle arrest and inhibited neural progenitor cells. This study supports the link between ZIKV infection and microcephaly, as it closely mimicked the physiology of infected human fetus of smaller brain size, a thinner cortical plate, enlarged lateral ventricle and ventricular/subventricular zones in the infected mouse brains (34). However, groundbreaking research of Tang et al. (35) on human neural Progenitor Stem cell (hNPCs) model establishes that ZIKV is a neurotropic virus that directly targets developing embryonic brain cells. ZIKV infection can increase cell death rate, it can further dysregulate cell-cycle progression and cause attenuated hNPC growth (35).

To mimic the ZIKV infection Rossi et al. (36) designed interferon (IFN) alpha receptors lacking mice models. They detected ZIKV in brain after 3 days post-infection and caused neurologic diseases after 6 days post-infection, indicating the neurotropic nature of ZIKV (36). ZIKV inoculation to pregnant mice resulted in infection of embryonic radial glial cells of the dorsal ventricular zone and reduction in lateral ventricles cavity (22, 36, 37). In a similar study, ZIKV injected to pregnant mice produced some animals with restricted intrauterine growth, stunted heads, and ocular anomalies (38). An experimental study resulted in placental infection, viral infection and reduced the number of dorsal and ventricular radial glial cells of brain, and viremia, when intraperitoneal inoculation of pregnant mice was performed (39).

### ZIKV Related *in-vitro* Studies

Researchers have successfully conducted experiments to investigate the ZIKV pathogenesis and its affinity for neurons and neural stem cells by using cellular models such as neural cell precursors (neurospheres), brain organoids, advance embryonic stem cell (ESC), induced pluripotent stem cell (iPSC) (12), spinal cord neuroepithelial stem (NES) cells, and neocortical cell models (40). Moreover, successful experiments on animal models such as mouse, rats, and nonhuman primates have broadened our understanding about ZIKV teratogenic effect on fetal brain.

Dang et al. (41) investigated the ZIKV and microcephaly relationship by using cerebral organoids derived human embryonic stem cells. Authors have demonstrated that ZIKV is capable to disturb cell fate and restrict organoids growth by disrupting the activation of innate immune receptor, Toll-like-Receptor 3 (TLR3). The activation of TLR3 pathway can alter genetic expression, disrupt neurogenetic pathways, affects apoptosis, and plays crucial role in microcephaly (41).

Garcez et al. (42) used human-induced pluripotent stem cells to investigate the ZIKV infection consequences on brain development in the first trimester with advance 3D culture models. The results illustrated that ZIKV can potentially cause death of human neural stem cells by declining the growth of brain organoids and neutrospheres. Qian et al. produced viral infections in a forebrain-specific organoid model derived from human-induced pluripotent stem cells, which resulted in microcephaly like symptoms, such as increased cell death, decline in cellular proliferation, and neuronal cell volume (43). For the mechanism of human brain injury, ZIKV is thought to reduce the formation of brain matter by affecting autophagy pathway, chromosomal stability, and centrosome segregation (44).

A more recent study has illustrated that the teratogenic effect and spectrum of fetal brain malformation induced by ZIKV is very broad and difficult to detect in clinical settings. In a research on pigtail macaque (Macaca nemestrina) nonhuman primate model, it is demonstrated that ZIKV can potentially target and destroy fetal neural stem cells, even if the baby is not microcephalic (40). Studies conducted on human astrocytes and glial cell lines support the previous research that ZIKV most likely infect neural stem cells, oligodendrocyte precursor cells, microglia and astrocytes, while neurons are less prone to get infected. Moreover, the macrolide antibiotic drug azithromycin can potentially protect brain cells from ZIKV infection by declining viral proliferation and its cytopathic effects in human astrocytes and glial cell lines (45).

## Challenges in ZIKV Related Studies

Despite the evidences supporting ZIKV association with microcephaly and neonatal/fetal brain congenital abnormalities, scientists from different backgrounds suggest that clinical symptoms of microcephaly are shared by various other congenital infections (e.g., cytomegalovirus), and arboviruses that can potentially cause microcephaly (31, 46). Furthermore, approximately 80% of ZIKV infected patients either had no symptoms or symptoms similar to that of DENV and CHIKV that were co-circulating during ZIKV outbreak in Brazil. Only 44% of reported cases were confirmed, while the remaining cases were considered normal, showing a degree of over-reporting (46). Presenting or lacking ZIKV like symptoms during pregnancy does not affirm that fetus will be microcephalic, even if this hypothesis is considered true the ratio of ZIKV related microcephaly cases are still ambiguous (1). An American reported case showed that pregnant women who got infected with ZIKV during 2nd and 3rd trimesters still delivered healthy babies (39). In certain Brazilian states, an analysis has estimated that 1st trimester of pregnancy has the highest risk of laboratory-confirmed ZIKV transmission and microcephaly as compared to 2nd and 3rd trimesters that have been linked with fetal death, defects in intrauterine growth, or abnormalities in prenatal imaging that needs to be confirmed postnatally as the pregnancies are still ongoing (31, 47). The retrospective study conducted by Cauchemez et al. estimated 1% risk of microcephaly to the fetuses/neonates, as discussed earlier, has provided crucial information about ZIKV, microcephaly, and brain anomalies (9). However, this study has drawbacks, such as estimation of risk based on small number of cases, wide confidence interval, and lack of control for other confounders (47).

Brazil is going through a severe economic recession and challenge to cope with the soaring unemployment among youth. The young people of child-bearing age having little or no income are going through serious economic-disadvantaged that is affecting their nutritional status directly that may add to a false diagnosis of microcephaly as malnutrition and can negatively impact fetal growth and head circumference (1, 5, 48). In Brazil, the diagnostic standard of microcephaly after December 2015 illustrating results with more specificity and sensitivity, pondered a question about the prevalence of microcephaly during 2015 (about 20 cases/10,000 births) as compared to 2014 (about 1 case/10,000 births) (49). Nutrition assessment and post-diagnosis follow-up is cautious and necessary for correctly diagnosing microcephaly ascribed to ZIKV because incorrect diagnosis may exaggerate, limit or reverse this relationship. Keeping in view the current uniform diagnostic standards, it is considered that the numbers of reported cases were over rated and falsely diagnosed, which were excluded in follow–up cohorts (39).

Ruling out other viral infections to confirm the ZIKV link with microcephaly is quite important (32), as during previous ZIKV epidemic in the Pacific Islands, no severe consequences during pregnancy were documented (47). ZIKV symptomatic mothers might be misdiagnosed as other viral diseases (11, 47), for instance, West Nile virus (WNV), Japanese encephalitis viruses, Dengue virus (DENV) and the yellow fever virus (46) that are genetically similar to ZIKV. ZIKV was not a reportable neurological disease until 2015, it was recently suspected to cause congenital disease in Brazil and no serological survey was done to confirm this link. Thus, a proportion can't be established on the basis of infected population in different geographical regions. Such information can only be used in predicting the course of a microcephaly outbreak (46). ZIKV epidemic in Cape Verde (2015–2016), probably caused by African strain mosquitoes, resulted in thousands of infection cases without causing any neurological disorder (20). Calvet et al. report on ZIKV genomic RNA detection by next-generation sequencing and quantitative RT-PCR is not enough as it only confirms that ZIKV is a cause of congenital infection (50), while no recombination events were indicated in the ZIKV sequenced genome, as genetic mutations for phenotypic changes has been reported previously for other closely related flaviviruses (1).

Since the exposure of ZIKV and its confirmation as a potential cause of microcephaly, there are few limitations reported in the studies, as discussed in detail by Wu et al. (39). There was a broad range of time interval (average, 13 weeks) since fetal microcephaly was first diagnosed, and maternal clinical symptoms appeared. In Brazil, the ZIKV epidemic could predict profound increase in the number of microcephaly cases, almost 5–10 months later. During this time maternal laboratory tests for ZIKV confirmation could be done in order to exclude any confounder as a nonspecific cause of microcephaly. Whereas, due to lack of official case by case reporting and a few numbers of laboratory confirmed cases, it is inappropriate to predict the total expected number of microcephaly cases in Brazil and in the rest of the Americas (39).

Brasil et al. claimed a positive association of ZIKV with microcephaly, i.e., 4/42 cases among the exposure group vs. 0/16 among the non-exposure group, without ZIKV laboratory confirmation and laboratory exclusion tests (31), for instance, plaque reduction neutralization test to minimize the chances of cross-reactivity of other vulnerable flaviviruses that can't be ruled out (1). The control group of these limited number of samples had a rash like symptoms during pregnancy, but the cause of rash was not analyzed or diagnosed clearly (39). Moreover, it is highly important to reexamine specimens from DENV epidemics and use of virus isolations or RT-PCR for laboratory diagnosis of DENV infections as an essential component of laboratory testing algorithms (51). If ZIKV is encountered as a first flavivirus the chances of cross-reactivity decreases; the result will be opposite if ZIKV causes a secondary flavivirus infection, because serological test results will be positive for DENV. If a population infected with ZIKV has a background immunity for DENV or any other flavivirus, DENV cross-reactivity IgM assay may lead to false results and misdiagnosed cases of ZIKV (51).

## Current and Future Implications of ZIKV

Above all, some important answers need to be explored in order to understand the emerging Zika virus (ZIKV), which remained obscure until the recent epidemic in French Polynesia (2013–2014) and in multiple countries of South America (2015–2016) (1). Evolutionary analyses have revealed that multiple mutations in ancestral Asian lineage are associated with the recent epidemics in the Americas. Clinical isolates collected from the recent ZIKV epidemic in the Americas were hyper infectious in mosquitoes as compared to the FSS13025 strain, isolated in 2010 in Cambodia. Surprisingly, recently isolated strains have evolved to attain an instinctive mutation in its Non-structural protein 1 (NS1) amino acid, resulting in high expression of NS1 antigenaemia. Higher NS1 antigenaemia expression in infected hosts promotes ZIKV infectivity and prevalence in mosquitoes, which could have facilitated transmission during recent ZIKV epidemics (52).

Similarly, a single amino acid substitution at serine to asparagine (S139N) in the viral polyprotein significantly increased ZIKV infectivity in both mouse and human neural progenitor cells (NPCs), resulting in microcephaly in the mouse fetus, and higher mortality in neonatal mice. Phylogenetic analyses have demonstrated that the S139N substitution arose before the French Polynesia outbreak the 2013 (1), was stably maintained during its subsequent propagation to the Americas. The functional adaption of ZIKV has made it more infectious to human NPCs, by contributing to the increased microcephaly rate in recent ZIKV epidemics (53).

Is it possible that genetic alterations of the virus have affected the replication mechanism, toxicity and its persistence in different geographical regions? What is the reason behind prolonged survival time of the virus in blood, brain tissues, amniotic fluids, and other tissues? How often infection status of the patients should be evaluated to respond the virus in a better way? Why developing brains provide more feasible micro-environment for the ZIKV? It is crucial to explore the full spectrum of abnormalities caused by ZIKV and solid reason of neurotropism. Although evidences are supporting ZIKV relationship with microcephaly and other brain injuries, yet information about its epidemiology, pathology and mechanism is evolving. Thus, we still need to answer aforementioned questions to address and understand unpredictable and rapidly growing ZIKV infection. It requires serious attention toward advance research, vaccine development and joint effort and collaboration between public and private sectors to look for innovative ways of ZIKV treatment and prevention.

It is essential to explore the association of ZIKV infection and microcephaly, especially keeping in view the 80% asymptomatic cases of ZIKV. Maternal clinical symptoms of ZIKV can validate the diagnosis, but fetal and maternal laboratory confirmation, fetal deformities, autopsies, and miscarriage products detection is prerequisite to provide solid evidence. Advance studies should be done to evaluate the persistence of ZIKV in asymptomatic men and prolonged genital shedding (54, 55), that may have implications for ZIKV genomic RNA detection (56). In pregnant women (symptomatic or asymptomatic), serum neutralizing antibody titers that are more than or equal to 4-fold higher than dengue neutralizing antibody can be a useful approach. Other arthritis causing infections causing such as, CHIKV, DENV, malaria, Rubella, Group A Streptococcus, Parvovirus, Leptospira, measles, and rickettsial infection should be eliminated (57).

At present no commercial test to diagnose Zika virus infection. The reverse transcription polymerase chain reaction (RT-PCR) is performed to detect ZIKV RNA in the laboratory. Antiviral immunoglobulin M (IgM) antibodies of ZIKV that appears at the end of the first week of onset of illness can be detected from the blood by either plaque reduction neutralization test (PRNT) or IgM enzyme linked immunosorbent assay (ELISA). Cross reaction of ZIKV with other Flaviviruses can be eliminated by using PRNT (57). However, restriction of these tests to a few specialized laboratories, the unavailability of these tests can be partially reduced by RT-PCR (9).

Already reported complicated epidemiological context of the concurrent circulating CHIKV, DENV, and ZIKV co-infections cannot be ignored. In French Polynesia, ZIKV infection was associated with autoimmune and neurological complications in the presence of co-circulating DENV (19). Further studies, genuine laboratory and clinical differential diagnostic tests among these infections is highly important to exclude the potential confounders, to unveil subsequent infections and co-infections by different arboviruses that can affect the course of the disease, their ways of transmission (vertical, perinatal, sexual) and the incidence of severe cases (5, 19, 48). The number of ZIKV related publications, guidelines, clinical cases, recommendations and circulating data (yet confirmed or not) is increasing day by day (1), therefore, standardized anthropometric applications, centralization of data (58), and advance experimental research is highly needed to confirm this relationship (9).

The populations should be well informed about the neurological malformations risks, especially in the regions where existence of Zika virus, its vectors or local transmission is suspected (20). Adequate counseling of ZIKV infected pregnant women and those preparing for pregnancy are advised to protect themselves from bites of mosquitos, avoid traveling to epidemic regions (9), and take precautions if their partners have returned from epidemic regions, or delay their pregnancies (59). Control and prevention strategies for ZIKV infection should also include increased use of insect repellent and other interventions to decrease the abundance of potent mosquito vectors (60). Epidemic countries or countries at higher risk should do a proper check up and go through follow-up cohorts and calculate potential risk of microcephaly throughout women pregnancy, along with proper investigation of materno-fetal transmission through experimental studies (9, 40).

The virological study of CHIKV can shed light on the isolated strains of ZIKV from Brazil, as the global epidemic and distribution of both viruses is similar. Phylogenetic analysis has shown that the Brazilian strains of ZIKV have evolved in a mixed way. A study suggested that isolated strains were closely related to Cambodian strain in 2010 (61), while presented 99% resemblance with French Polynesian strain in 2013. Brazilian Zika virus strains coming from Asian lineage had alteration in 6–15 amino acids as compared to the French Polynesian strain (30).

Despite the tremendous novel work is now underway to develop ZIKV vaccines, the important challenges still remain for the preclinical and early clinical studies. It is suggested that previous successful licensed viral vaccines experience of other flaviviruses (such as YFV, JEV, and DENV) (62), and use of currently available preclinical ZIKV vaccine data can pave road to develop effective ZIKV vaccine. ZIKV clinical vaccines development is efficient, focused and uniquely collaborative, while numerous phase 1 clinical trials have been initiated within the first year of ZIKV epidemic (63). Recombinant DNA technology that plays an important role in improving health conditions by developing new pharmaceuticals, vaccines, monitoring devices, diagnostic kits, and therapeutics (64) should be considered to clinically develop ZIKV vaccine. Perhaps this technology can help to design a targeted antiviral vaccine, and thoroughly explain the ZIKV molecular interaction with the hosts (58). Specially, DNA and purified inactivated virus (PIV) vaccines are more attractive for ZIKV infection by keeping in view the theoretical safety benefit of non-replicating vaccines in women. Use of neutralizing antibodies is also supposed to play a pivotal role in vaccine development against ZIKV infection in future (63).

### Nanotechnology and Zika Virus: Treatment and Diagnosis

Nanotechnology is used for the diagnosis of Zika virus (ZIKV) and dengue virus (DENV) infections; multiplexed assay on a plasmonic-gold (pGOLD) platform was developed for the exact measurement of IgG, IgA antibodies and IgG avidity in the blood serum of the patients. These antibodies (IgA and IgG) are more specific against NS1 antigen of ZIKV infection in comparison to IgM cross reactivity method (65).

Nanopharmaceutical represents any nanomaterial having therapeutic potential that can be used as a therapeutic agent for example, liposomes, dendrimers, micelles, and nanocapsules. Nanoparticles can have different chemical compositions and shapes, and it can be categorized according to characteristics of matrix or the method drug delivery (66). Up till now there is no available drug or a vaccine in market that is effective against ZIKV infection. However, one pharmaceutical company demonstrated clinical trials with nano-based antiviral drug have shown some potential against ZIKV. Basically, this drug (trade name: VivaGel^®^) is Lysine-based dendrimer with naphthalene disulfonic acid surface groups that can be potentially used against sexually transmitted herpes simplex virus (HSV), human immunodeficiency virus (HIV), and human papillomavirus (HPV); and is expected to show some antiviral activity against ZIKV in trials (67, 68). Different antiviral nano-medicines have different modes of action, thus there is a possibility to design and check the effectiveness of the similar antiviral nanomedicines against ZIKV in near future. For example, inactivated virosomal (liposome) and virosomal (liposome) vaccine is used for the treatment of influenza and hepatitis A virus (HAV) (69–71). Similarly, another therapeutic vaccine for HIV is under clinical trials that contains synthetic plasmid DNA immunogen and induces expansion of the HIV-specific precursor (72). Another example is use of wet lipid nanoparticles against hepatitis B virus (HBV) that target 3 sites on the genome of HBV was designed using lipid particle coupled with 3 RNAi therapeutics (73–75). Moreover, for HIV, solid drug nanoparticle formulation is at clinical trials; basically, it is non-nucleoside reverse transcriptase inhibitor (76, 77). In future there is a possibility of designing such kind of the antiviral nanomedicines with different mechanism of action which may show positive outcomes to treat ZIKV.

## Conclusions

Apart West Nile virus (78), thousands of congenital microcephaly cases, fetal brain tissue damage and neurological syndromes have been associated with ZIKV infection. Unfortunately, the epidemics of this mosquito born, and a relative stable virus is on a rise. Although congenital microcephaly is a rare disorder however, due to lack of standardized diagnostic test facilities, the incidence in the geographically widespread ZIKV epidemic regions is higher. Animals studies showed that ZIKV is a neurotropic virus. It directly targets the developing embryonic brain cells by inducing apoptosis, cell-cycle arrest, and dysregulate hNPCs. During pregnancy in mice, ZIKV inoculation resulted in stunted heads, restricted intrauterine growth, and ocular anomalies. Despite this, still, the information about its epidemiology, pathology and mechanism is not clear. Therefore, further studies are needed toward advance research and vaccine development for ZIKV treatment and prevention.

## Author Contributions

RS conceived and wrote the manuscript. YL and WS edited the manuscript. GN critically reviewed and edited the manuscript. MX and SK revised, supervised, and funded the study.

### Conflict of Interest Statement

The authors declare that the research was conducted in the absence of any commercial or financial relationships that could be construed as a potential conflict of interest.

## Abbreviations

ZIKV: Zika Virus
HC: Head Circumference
SD: Standard Deviation
DENV: Dengue Virus
CHIKV: Chikungunya Virus
WNV: West Nile Virus
YFV: Yellow Fever Virus
purified inactivated virus (PIV)
MRI: Magnetic Resonance Imaging
RT-PCR: Reverse-transcription-polymerase Chain Reaction
PRNT: Plaque Reduction Neutralization Tests
ELISA: Enzyme-linked Immunosorbent Assay.
